# Eyes As the Window to Syphilis: A Rare Case of Ocular Syphilis As the Initial Presentation of Syphilis

**DOI:** 10.7759/cureus.6998

**Published:** 2020-02-14

**Authors:** Shweta Paulraj, Prashanth Ashok Kumar, Harvir Singh Gambhir

**Affiliations:** 1 Internal Medicine, Upstate Medical University, Syracuse, USA

**Keywords:** neurosyphilis, ocular syphilis, scotoma, black spot, syphilis, hiv, rash, penicillin

## Abstract

In the pre-antibiotic era, neurosyphilis (NS) was common, occurring in 34% of patients with syphilis. Currently, there has been a rising trend in syphilis with HIV-infected patients being more prone to develop NS. Ocular involvement is very rare in NS and accounts for only 1%-5% of the cases in the United States. We report the case of a 53-year-old male with a past medical history of gastroesophageal reflux disease and hyperlipidemia who presented to his ophthalmologist for blurred vision in both eyes. He had been noticing a black spot in the visual field of his left eye for two weeks. He had also noticed a rash on his forearms. His past and social history was significant for treated Lyme disease, having pet cats. He identified as a heterosexual male, married, and with five children. However, on further history taking, he reported a homosexual exposure about five years prior. He denied any history of genital ulcer or penile discharge. On examination at the ophthalmology clinic, he was found to have a visual acuity of 20/20 right eye and 20/100 left eye. Posterior segment examination of the both eyes showed subtle neuritis and vasculitis. Fundus photography revealed subtle neuroretinitis bilaterally. Work up was initiated for inflammatory and infectious causes. His rapid plasma reagin and fluorescent treponemal antibody absorption showed positive titers for syphilis. His presentation was most consistent with ocular syphilis. A lumbar puncture (LP) was done with Venereal Disease Research Laboratory (VDRL) positivity in the spinal fluid. He was therefore initiated on intravenous (IV) penicillin four million units every four hours for 14 days. His ophthalmology follow-up after one month showed both subjective and objective improvement in his visual symptoms. He also followed with the infectious disease team and a repeat LP done three months later showed nonreactive VDRL in cerebrospinal fluid (CSF).

Ocular syphilis is increasing in incidence. Clinical presentation is variable, and a high index of suspicion with a low threshold for serological testing are important as early treatment can reverse retinal changes and restore visual acuity. There is a recommendation for CSF examination in all patients with ocular syphilis including HIV-negative cases. There have been studies showing a high CSF abnormal rate in HIV-negative patients with ocular syphilis. The recommended treatment for NS is aqueous crystalline penicillin G (18 to 24 million units per day, administered as three to four million units IV every four hours, or 24 million units daily as a continuous infusion) for 10 to 14 days. Follow-up is a key component of management with neurological examination and LP for CSF VDRL performed three months after treatment and every six months after, until the CSF is nonreactive for VDRL with normal white blood cell count.

It is important to be cognizant of the rising trend of ocular syphilis, even in HIV-negative individuals. Early treatment is time sensitive to preventing permanent vision loss. Our case also emphasizes on thorough history taking, even for patients who appear to be at a low risk for sexually transmitted infections.

## Introduction

Syphilis is an ancient, chronic, sexually transmitted disease caused by Treponema pallidum, with a spectrum of presentations [1]. In the pre-antibiotic era neurosyphilis (NS) was common, occurring in 34% of patients with syphilis [2]. With the discovery of penicillin, there had been a steady decline in the incidence of syphilis. However, there is currently an almost universal trend of rise in the incidence of syphilis among men who have sex with men [3,4]. HIV-infected patients in particular are more prone to develop NS [5]. Ocular involvement is very rare in NS and accounts for only 1%-5% of the cases in the United States [6,7]. We report the case of a patient who presented with a "black spot" in his vision and was found to have ocular syphilis and NS. We emphasize on a renewed awareness of this condition to facilitate early detection and timely management.

## Case presentation

**Figure 1:**
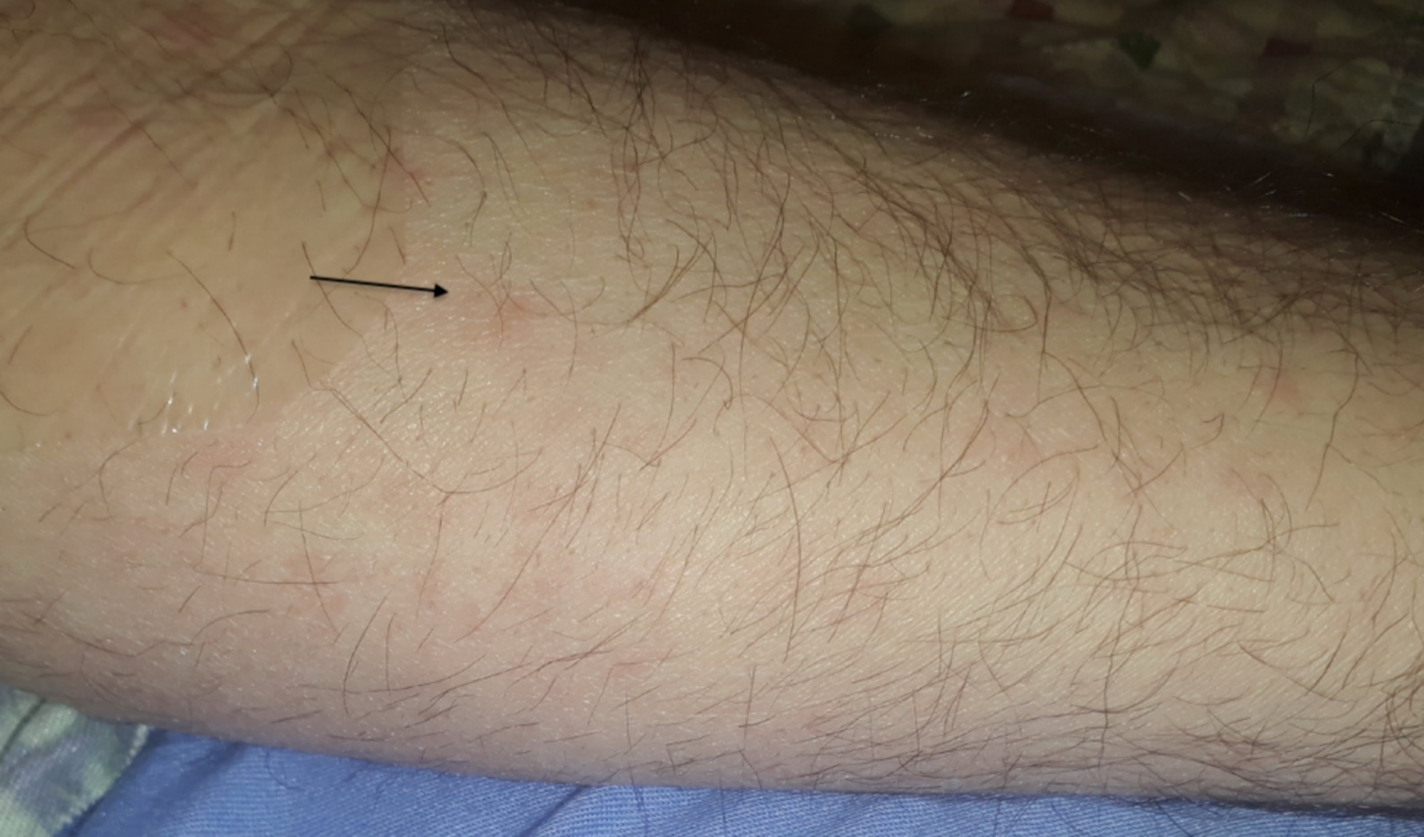
Maculopapular rash on forearm suggestive of secondary syphilis Arrow points to rash

We report the case of a 53-year-old male with a past medical history of gastroesophageal reflux disease and hyperlipidemia who presented to his ophthalmologist for blurred vision in both eyes. He had been noticing a black spot in the visual field of his left eye for about two weeks. He also had a rash on his forearms and chest which he had noticed around the time of his visual changes (Figure 1).

**Figure 2:**
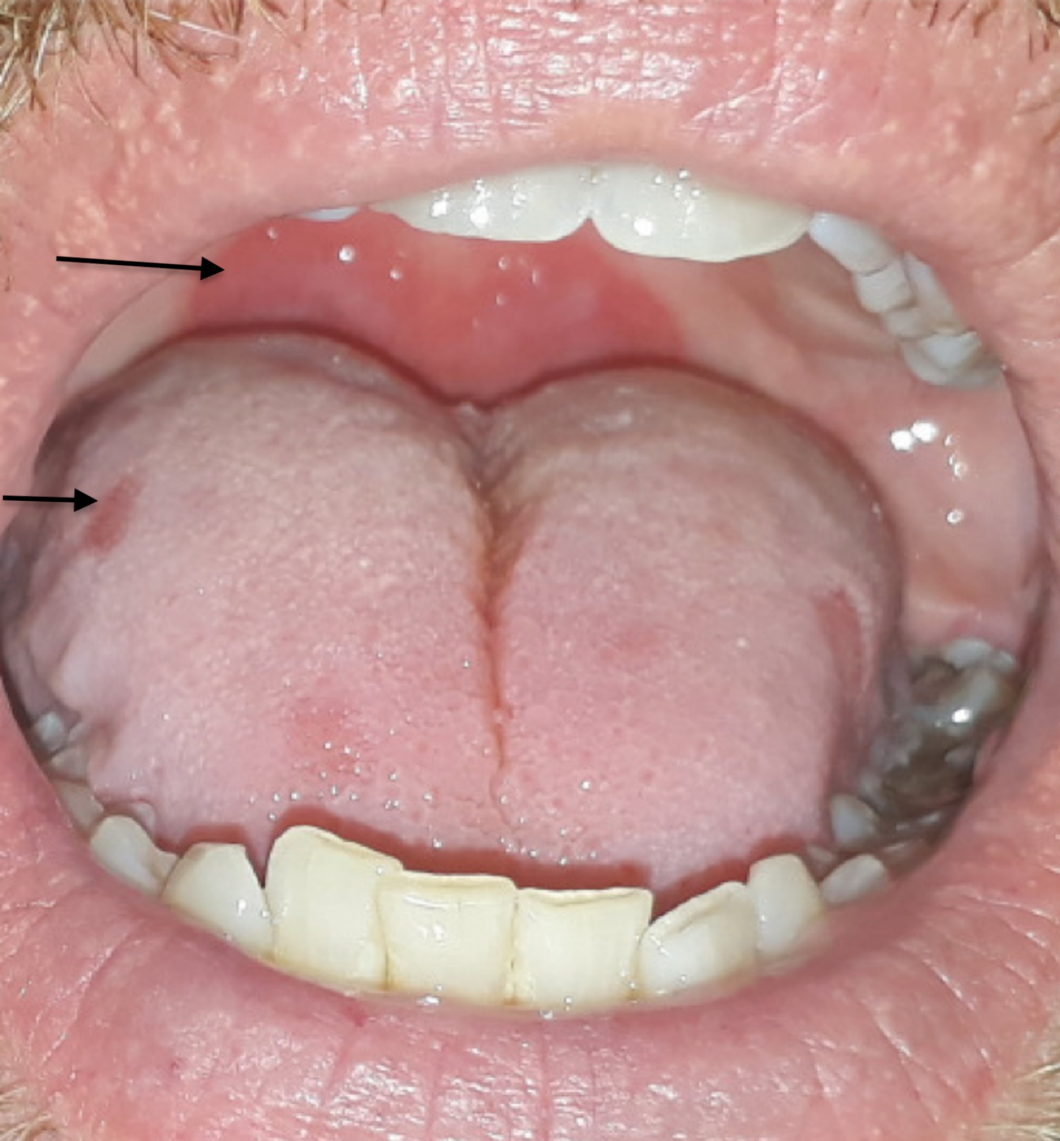
Oral mucosal lesions Arrows point to erythematous mucosal lesions

He denied any fevers, chills, weight loss, appetite changes, nausea, vomiting, diarrhea, or joint pains. He denied any history of genital ulcer or penile discharge. He had a history of Lyme disease which had been treated in the past. He currently had cats at home. With regard to his sexual history, he identified as a heterosexual male, denied high-risk sexual behavior, and was married with five children. However, on further history taking, he reported a homosexual encounter about five years prior. He was unsure about his HIV status and had never tested positive for HIV in the past. On initial examination, he was afebrile with stable hemodynamics. It was noticed that he also had erythematous lesions in his oral mucosa (Figure 2).

**Figure 3:**
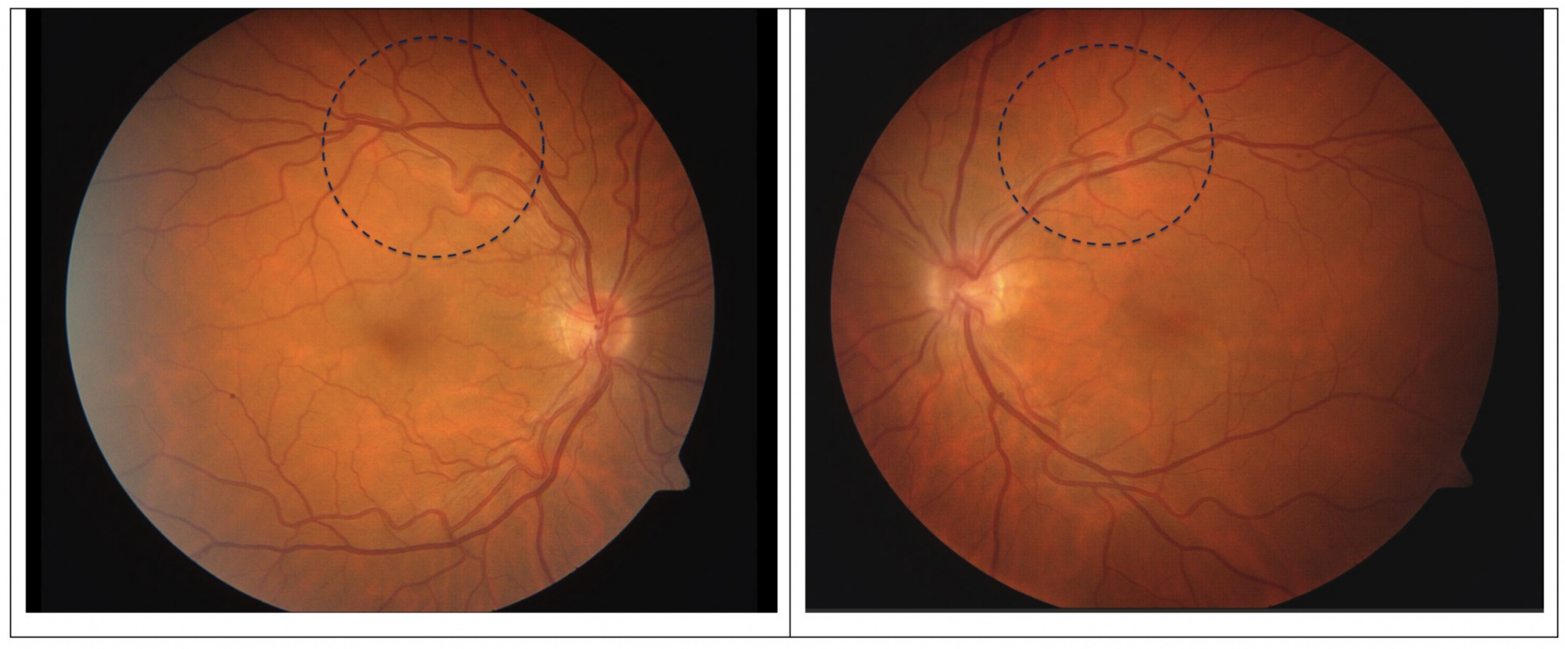
Right and left fundus photography From left to right: Right fundus and left fundus photographs. Dotted circles represent areas of superotemporal vasculitis with disrupted flow

His visual acuity was 20/20 in his right eye and 20/100 in his left eye. Bilaterally, the anterior segments showed a clear cornea, deep and quiet anterior chambers, and round iris with a clear lens. The posterior segment examination of the both eyes showed an unattached posterior hyaloid and subtle fullness of optic nerve. Subtle neuritis and vasculitis were seen bilaterally with vitreous cells in the left eye. Fundus photography was done and revealed subtle neuroretinitis bilaterally, more so in the left eye (Figure 3).

Evaluation for infectious and inflammatory causes of neuroretinitis was initiated including Bartonella antibodies, Lyme antibodies, and Treponema pallidum testing. His rapid plasma reagin (RPR) testing was positive with titers at 1:128. Fluorescent treponemal antibody absorption (FTA-Abs) testing was also positive. Testing for other sexually transmissible diseases such as HIV and hepatitis was negative. He was diagnosed to have ocular syphilis and a lumbar puncture was done showing Venereal Disease Research Laboratory (VDRL) positivity in the cerebrospinal fluid (CSF). He was subsequently treated with intravenous penicillin G, four million units every four hours for a total of 14 days. He was closely followed up by the infectious diseases team and the ophthalmology team. His visual symptoms improved subjectively and objectively on follow-up with ophthalmology. Repeat lumbar puncture was done in three months and showed nonreactive VDRL and white blood cell (WBC) count of <3 . He is planned for a lumbar puncture every six months until the WBC count in his CSF normalizes and repeats RPR every six months for one to two years

## Discussion

Ocular syphilis can occur at any time in the course of syphilis but often accompanies early NS with acute meningitis. NS by itself can sometimes be asymptomatic, presenting without meningitis and diagnosed by serological and CSF criteria [8]. Ocular syphilis has a myriad of manifestations, typically in secondary and tertiary syphilis. The most common manifestations are panuveitis or posterior uveitis, followed by interstitial keratitis, chorioretinitis, neuroretinitis, retinal vasculitis, and neuropathies [9]. The clinical presentation of neuroretinitis is usually of decline in visual acuity followed by central or cecocentral scotomas [10]. This was identical to the presenting symptoms in our patient with a subsequent diagnosis of neuroretinitis on ophthalmological evaluation.

Currently, due to the common co-occurrence of syphilis in HIV patients, there is a low index of suspicion in HIV-negative individuals. As per Kunkel et al., ocular syphilis is less common in HIV uninfected healthy patients as compared to those with HIV infection [11]. However, as highlighted by our case, a high index of suspicion and a low threshold for serological testing are important as early treatment can reverse retinal changes and restore visual acuity. Early treatment in patients with suspicious uveitis usually has good outcomes with regard to visual changes [12]. There have been studies showing a high CSF abnormal rate in HIV-negative patients with ocular syphilis [13]. In our HIV-negative patient, CSF examination did reveal concomitant syphilitic meningitis. This reinforces the recommendation of CSF examination for all patients with ocular syphilis including HIV-negative cases [14]. Treatment delays with ocular syphilis can lead to permanent visual loss secondary to atrophy of the optic nerve and retina [15]

The diagnostic modality for any patient with suspicion for syphilis is serological testing commonly with RPR testing and FTA-Abs testing to initially confirm the diagnosis. For patients with syphilitic meningitis, a reactive CSF VDRL confirms the diagnosis, although it cannot be ruled out by a nonreactive test [16]. Treatment of ocular syphilis is aqueous crystalline penicillin G 18 to 24 million units per day, administered as three to four million units intravenously every four hours, or 24 million units daily as a continuous infusion for 10 to 14 days. Regardless of the results of CSF examination, ocular syphilis should be treated as per the recommendations for NS [17]. In patients with CSF abnormality, follow-up is a key component of management with neurological examination and lumbar puncture for CSF VDRL performed three months after treatment and every six months after, until the CSF is nonreactive for VDRL with normal WBC count [18]. In addition, RPR titers are monitored every six months until there is a fourfold decline in the titer. Most but not all patients may undergo seroversion [19].

## Conclusions

It is important to be cognizant of the rising trend of ocular syphilis, even in HIV-negative individuals. A high index of suspicion and early treatment are time sensitive to preventing permanent vision loss. Our case also emphasizes on thorough history taking, even for patients who appear to be at a low risk for sexually transmitted infections.
